# The future-focused Proactive Conservation Index highlights unrecognized global priorities for vertebrate conservation

**DOI:** 10.1371/journal.pbio.3003422

**Published:** 2025-10-21

**Authors:** Gabriel Henrique de Oliveira Caetano, Gopal Murali, Daniel Pincheira-Donoso, Reut Vardi, Lior Greenspoon, Shai Meiri, Uri Roll

**Affiliations:** 1 Université Paris-Saclay, CNRS, AgroParisTech, Ecologie Systématique et Evolution, Gif-sur-Yvette, France; 2 Jacob Blaustein Center for Scientific Cooperation, The Jacob Blaustein Institutes for Desert Research, Ben-Gurion University of the Negev, Midreshet Ben-Gurion, Israel; 3 Mitrani Department of Desert Ecology, The Jacob Blaustein Institutes of Desert Research, Ben-Gurion University of the Negev, Midreshet Ben-Gurion, Israel; 4 Department of Ecology and Evolutionary Biology, University of Arizona, Tucson, Arizona, United States of America; 5 Center for Ecological Sciences, Indian Institute of Science, Bangalore, Karnataka, India; 6 School of Biological Sciences, Queen’s University Belfast, Belfast, United Kingdom; 7 School of Zoology, Tel Aviv University, Tel Aviv, Israel; 8 School of Geography and the Environment, University of Oxford, Oxford, United Kingdom; 9 Department of Plant and Environmental Sciences, Weizmann Institute of Science, Rehovot, Israel; 10 School of Biosciences, University of Melbourne, Parkville, Victoria, Australia

## Abstract

Human-induced environmental pressures are expected to intensify worldwide during the 21st century. Consequently, future-focused tools and approaches to anticipate pressures on biodiversity are key to effectively prioritize conservation actions and supplement existing approaches. Here, we develop a continuous conservation prioritization index, the Proactive Conservation Index (PCI), that integrates projected future extrinsic threats and traits that can predispose species’ vulnerability. We used the PCI to assess the conservation priority of 33,560 species of land vertebrates worldwide, compared our results to the extinction risk categories of these species in the International Union for Conservation of Nature (IUCN) Red List of Threatened Species, and examined spatial and phylogenetic patterns in these species future conservation needs. We found that median PCI scores broadly followed the order expected under the IUCN Red List classification, but varied substantially within each IUCN Red List category. According to the PCI, reptiles will be the group of land vertebrates with highest conservation priority in the future, despite amphibians currently having the highest proportion of threatened species according to the IUCN Red List. The PCI revealed that species in the Near Threatened category will have future conservation needs more similar to species in threatened categories than to species in the Least Concern category. Arid ecoregions, tropical montane forests, and islands showed the highest differences between conservation priorities set using the PCI and the IUCN Red List, indicating possible unrecognized future conservation needs. The proportion of threatened species according to the IUCN Red List was uncorrelated with the protected area coverage of each ecoregion, while the PCI, by design, highlighted currently unprotected ecoregions with sensitive fauna that will have high exposure to threats in the future. We produced a user-friendly web application to display our results and an R package to enable users to calculate PCI scores for any taxon and region, customizing the index according to the severity of predicted threats and importance of species attributes in other systems. Our novel index can help practitioners prioritize fine-scale species conservation actions in light of future threats and different global change scenarios.

## Abstract 2

Human-induced environmental pressures are expected to intensify worldwide during this century. This study presents the Proactive Conservation Index, a new tool that allows fine conservation prioritization regarding future threats for any group of species, and applies it to all terrestrial vertebrates globally.

## Introduction

Human-induced climate, land, and sea use changes, organism exploitation, and expansion of invasive species are causing population and species extinctions at an unprecedented rate [1]. These processes are expected to intensify during this century [2–4]; e.g., 41% of land vertebrates are expected to have most of their ranges subject to unprecedented climate extremes by 2100 [5], and land-use change will cause land vertebrates to lose up to 11% of their current range every decade [6]. Such threats to biodiversity are driven by the expanding human population [7], which is projected to undergo a net increase of 30% by 2100 [8], along with an increase in per-capita resource consumption [9].

The vulnerability of species to these threats can be affected by their range size, conservation efforts they receive, and specific organism traits [10]. For example, large-bodied mammals, birds, and reptiles have been shown to face higher extinction risk, while the reverse was found in amphibians, with small-bodied species facing higher risks [11,12]. Additionally, many studies highlighted low reproductive rates as a key factor predisposing species to extinction (e.g., [13,14]). Thus, it is important for conservation prioritization schemes to also consider the diverse ways in which organisms are sensitive to threats.

The International Union for Conservation of Nature’s (IUCN) Red List of Threatened Species [3] (henceforth “IUCN Red List”) is the most widely used assessment of species extinction risk [15]. The IUCN Red List has been instrumental in guiding scientific research and conservation actions [16]. As of November 2024, 166,061 species have been assigned a threat category by the IUCN Red List [3]. One-fifth of assessed land vertebrate species are designated by the IUCN Red List as threatened, but over 22,000 species (~13% of all assessed species) are classified as Data Deficient (DD) [3]. This excludes them from conservation prioritization schemes based on IUCN Red List categories. Furthermore, most of the IUCN Red List’s assessments rely on past or current declines in population sizes or in extent or quality of distribution range [17,18]. Future effects of climate and land-use changes are seldom incorporated into most species-specific conservation prioritization lists [18]. Many studies, however, have found important differences between current and future threats, and emphasized the importance of more proactive conservation efforts [19,20].

**Fig 1:**
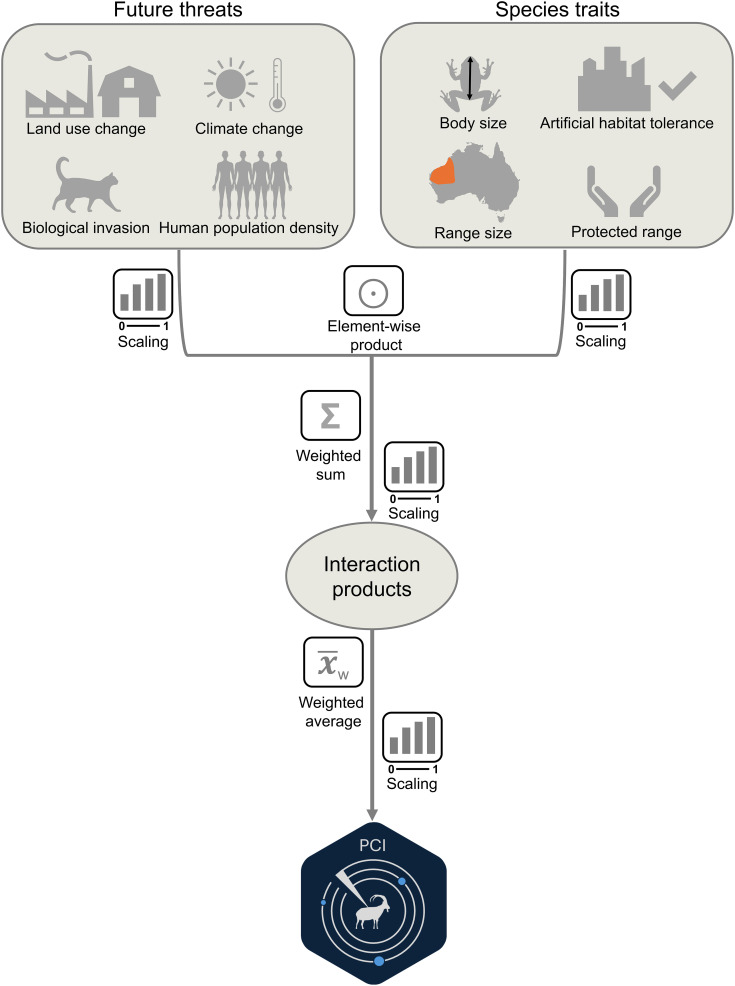
Proactive Conservation Index (PCI) calculation flowchart.

Here, we propose the Proactive Conservation Index (PCI), a novel conservation prioritization tool that is focused on future threats [21–23]. The PCI explicitly incorporates the role that forecasted drivers of biodiversity loss are expected to play in the future (human population increase, climate change, land use changes, and increases in biological invasions), as well species-specific attributes which modulate their vulnerability to these threats (body size, brood size, range size, intolerance to artificial habitats, and protected area coverage; Fig 1). The PCI is continuous, ranging from 0 (lowest conservation priority) to 1 (highest conservation priority), providing a nuanced picture ranking future conservation prioritization needs of species, rather than relying on past threats only. The PCI further enables conservation practitioners to assign different importance to each threatening attribute and can be calculated with readily available data. Thus, the PCI offers a method that can be applied to a wide range of taxa, including species classified as Data Deficient and those not yet evaluated in the IUCN Red List, which cannot be included in other conservation prioritization schemes. It further allows instant evaluation of thousands of species under different future scenarios.

To exemplify its utility, we calculated PCI scores for all land vertebrate species. The PCI is designed as a future-focused prioritization tool, not as a direct measure of extinction risk. Because the IUCN Red List is often used for conservation prioritization [16], despite being an extinction risk framework, we include comparisons with it to illustrate how the PCI can provide complementary insights. Thus, we emphasize that the PCI is best interpreted as complementary to, rather than a replacement for, the IUCN Red List assessments. We uncovered spatial and phylogenetic patterns of species conservation needs in different climatic and socioeconomic future scenarios (shared socioeconomic pathways [SSP, 24]) for the years 2050 and 2100. Furthermore, we provide an online application and statistical package for the software R [25] to easily enable the visualization of our results and the implementation of the PCI.

## Results

### Complementarity of the PCI and the IUCN Red List

**Fig 2:**
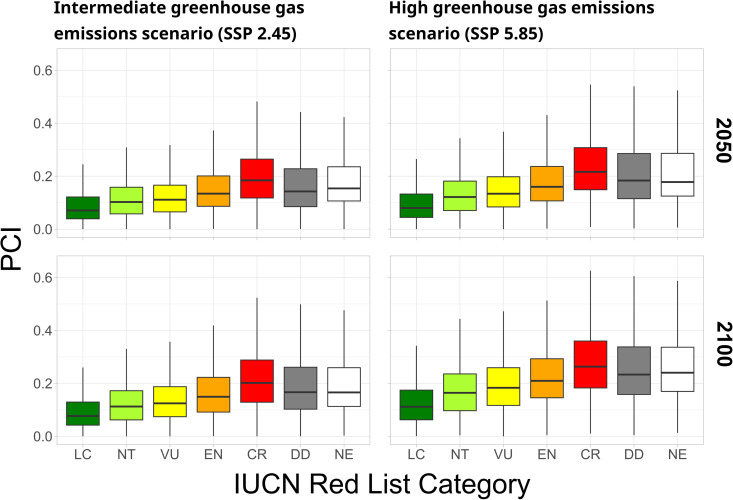
Distribution of Proactive Conservation Index (PCI) scores for all land vertebrates for the years 2050 and 2100, under two different greenhouse gas emission scenarios, compared to current IUCN Red List categories. Shared Socioeconomic Pathways (SSP) are greenhouse gas emission scenarios provided by the Intergovernmental Panel on Climate Change [24]. The data underlying this figure can be found in https://zenodo.org/records/17080841.

PCI scores varied substantially across species assigned identical IUCN Red List categories (Fig 2, standard errors range: 0.07–0.13 on a 0–1 scale). Nevertheless, PCI scores were significantly correlated to ordinated IUCN Red List threat categories under all future scenarios and years (Spearman’s rho ranging from 0.32 to 0.37, depending on which future scenario, S1 Table). To assess if this correlation is higher when including only species assessed under criteria which explicitly consider future projections, we repeated this analysis using only species categorized as threatened under criteria A3 and E, which are solely based on future threats (310 species), plus all non-threatened species. For this subset of species, the correlation between the PCI and the IUCN Red List was weaker than with all species, but still significant (Spearman’s rho ranging from 0.13 to 0.16, S1 Table). This suggests our approach to assess future conservation priority provides additional information to the methods used to assess the very few species listed as threatened under categories A3 and E.

Across all species analyzed, Non-Evaluated and Data Deficient species’ PCI scores were similar to those of Endangered or Critically Endangered species (Fig 2). Moreover, species classified as Near Threatened by the IUCN Red List had PCI scores more similar to species in threatened categories than to species in the Least Concern category (Fig 2). All results are based on the equal weight for all threats and traits used in the index calculation, except for results explicitly demonstrating the effects of variation in weighting (S1 and S2 Figs). Assigning higher weights to climate change when calculating PCI scores increased their correlation to ordinated IUCN Red List categories, and assigning higher weights to land use change, biological invasions, body mass, or brood size reduced this correlation, for all future scenarios (S1 Fig).

### Phylogenetic and geographic patterns

Under all future scenarios, reptiles had the highest median PCI scores, followed by mammals and amphibians, while birds had the lowest median scores (S3 Fig and S2 Table). We compared the distribution of average PCI scores and the proportion of threatened species according to the IUCN Red List in each family, for all classes together (S1 Data). 38% of families fall in the same quartile in both prioritization methods. We identified the families that are in the highest quartile for one prioritization method and in the lowest quartile for the other, to identify the taxonomic complementarity of both methods. These were mostly species-poor families, with 70% of them having five species or less (S1 Data). Among the families with high PCI scores and low proportion of threatened species are fossorial reptiles and amphibians, desert mammals, and island birds (S1 Data and S4–S7 Figs).

**Fig 3:**
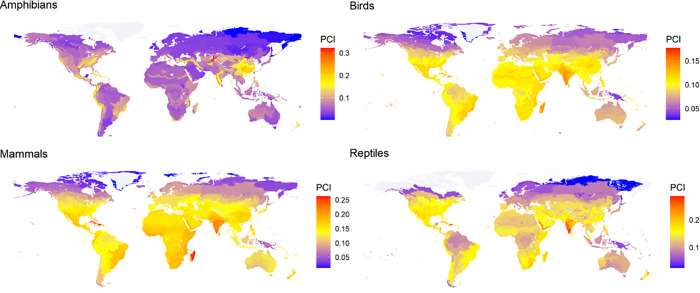
Average Proactive Conservation Index (PCI) scores for four land vertebrate classes, across ecoregions of the world, in the year 2100 under a high greenhouse gas emission scenario (SSP 5.85). Shapefile for ecorregions was obtained from Olson and colleagues 2001 [75]. The data underlying this figure can be found in https://zenodo.org/records/17080841.

Broad geographical patterns in average PCI scores per ecoregion were consistent across most scenarios (S8 Fig) and increased mostly in tropical ecoregions under SSP 5.85 in 2100. We focus on SSP 5.85 in 2100 for our subsequent results and discussion of geographic patterns (results for other scenarios can be found in S8 Fig). When considering the average of all classes, arid shrublands in the island of Socotra had the highest PCI scores, followed by high altitude forests in western India and insular tropical forest ecoregions in the Caribbean. These regions were consistently the highest for individual classes, with some important exceptions. Amphibians did not have high scores for islands but had high scores for forests in China and Central Asia. Mammals also had very high scores for Madagascar. For birds, high score islands were mostly in Polynesia, instead of the Caribbean. Average PCI scores for each class in each ecoregion are found in Fig 3 and S2 Data.

**Fig 4:**
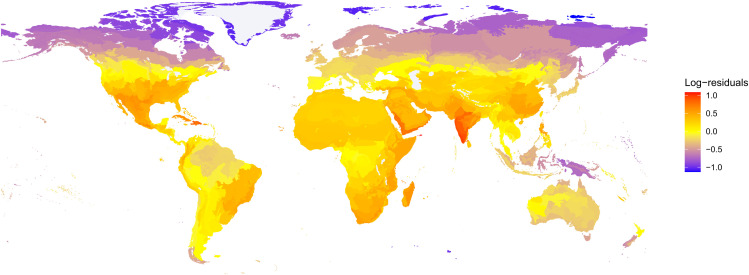
Log-transformed residuals of generalized linear model between the proportion of threatened species according to the IUCN Red List and average Proactive Conservation Index (PCI) score for each ecoregion of the world for all land vertebrates combined. Regions where the residuals are high (yellow to red) are projected to be more threatened by future threats than current IUCN assessments suggest. Shapefile for ecorregions was obtained from Olson and colleagues 2001 [75]. The data underlying this figure can be found in https://zenodo.org/records/17080841.

The proportion of land vertebrates in threatened IUCN Red List categories for each ecoregion of the world was positively correlated with the average ecoregion PCI scores (PCI effect: 1.971, error 0.970, *z*-value: 2.031, *p*-value 0.04). We examined the residuals of this regression to identify regions where the PCI predicts higher conservation priority than expected by the IUCN Red List (Fig 4). These regions were mostly consistent with high PCI score regions discussed above, including Socotra, West India, and the Caribbean. However, arid and semiarid regions around the world emerge as having high PCI scores and low proportion of threatened species. Residuals are especially large in the Arabian Peninsula and northern Mexico (Fig 4 and S3 Data). The proportion of threatened species in an ecoregion was uncorrelated with the ecoregion’s protected area coverage, but this proportion was strongly negatively correlated with average PCI scores (Spearman’s rho: −0.29, *p* < 0.001). This result is expected, since the PCI incorporates protected area coverage in its calculation.

Median PCI scores increased when assigning higher weights to land use change and artificial habitat intolerance, and decreased when assigning higher weights to climate change, biological invasions, human population density, protected range area, and geographic range size (S2 Fig). This pattern was consistent for most future scenarios, except for the 2100 SSP 5.85 scenario, in which increasing the weight of climate change increased PCI scores, and land use change made no difference (S2 Fig).

We identified four clusters of species (S9 Fig), characterized mostly by differences in their range areas and in levels of change in climate, land use, and human population density they will experience (S10 Fig). The distribution of threat variables between clusters was consistent across future scenarios, but in the year 2100 under the SSP 5.85 scenario, climate change contribution rose substantially for all clusters (S10 Fig). Amphibians and reptiles were proportionally more represented in the third and fourth clusters, which were most influenced by climate change (S11 Fig).

### Online resources for implementation

We developed a free online web application (using the R Shiny package; [26]) to display PCI scores for land vertebrates, including threats affecting each species and attributes that may increase their vulnerability to these threats (https://gabrielhoc.shinyapps.io/pci_app/). We additionally created an R package (https://github.com/gabrielhoc/PCI), that allows quick calculation of PCI scores for any taxon. Any variable (external threats or species attributes) can be used to calculate the PCI using this package, and different weights can be assigned to each variable. This enables tailoring the PCI for specific species groups, local contexts, or data availability.

## Discussion

We present a novel index to assess future conservation priorities for wildlife in an age of rapidly expanding anthropogenic pressures on natural environments and on the persistence of biodiversity globally. The PCI offers several advantages: (a) it focuses on projected future threats for species, (b) its continuous scale can enable a nuanced species prioritization, (c) it can be calculated quickly and using widely available data, and (d) it can be easily tailored to species groups and local contexts. Our implementation of the PCI for land vertebrates reveals that: (a) there is substantial variation in future conservation needs for species grouped under the same IUCN Red List category, (b) species classified as Data Deficient, Non-Evaluated, or Near Threatened, are projected to have high conservation priority in the future, (c) species in arid regions, tropical islands, and tropical montane forests, especially reptiles, are projected to have high conservation priority in the future than currently recognized, and (d) climate change mitigation and expansion of the protected area network can substantially alleviate effects of future threats.

### Complementarity of the PCI and the IUCN Red List

**Fig 5:**
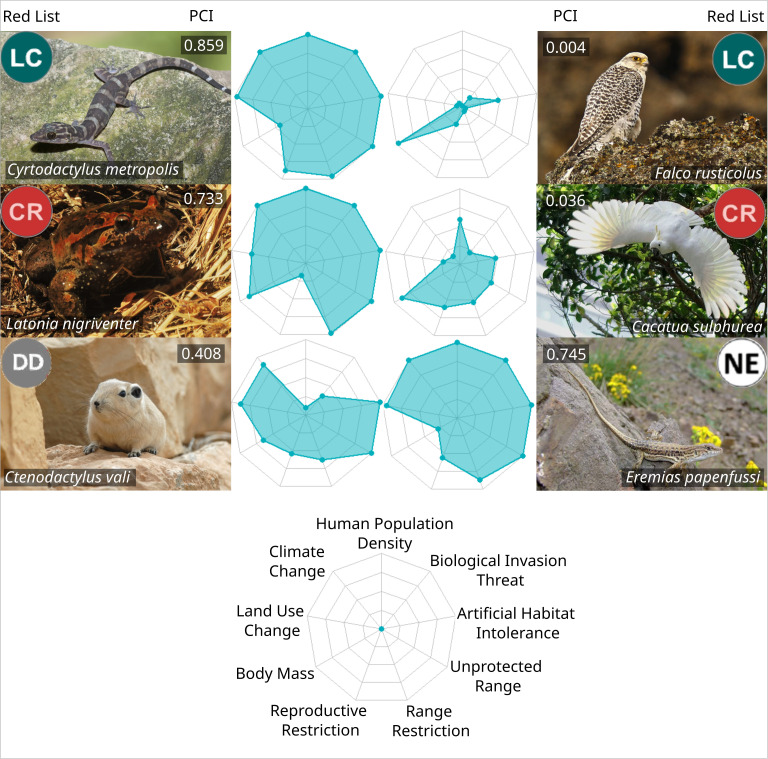
Distribution of variables used in the calculation of Proactive Conservation Index (PCI) for six species of land vertebrates. Values in spider plots were scaled between 0 and 1. Values on the upper part of each photograph indicate our assigned PCI scores (inner, values closer to 1 indicate a higher conservation priority, relative to other species in the same class), and their IUCN Red List categories (outer, LC, Least Concern; CR, Critically Endangered; DD, Data Deficient; NE, Non-Evaluated). To keep the area of spider plots proportional to PCI scores, we have inverted variables that reduce PCI score, and changed their names here accordingly: inverted brood size is named “Reproductive Restriction,” inverted range size is named “Range Restriction,” and inverted protected range is named “Unprotected Range.” *Cyrtodactylus metropolis* photograph provided by L. Lee Grismer, *Latonia nigriventer* image by UR, all other photographs obtained from Wikimedia Commons (authors: Momofelit, Ólafur Larsen, Charles Lam, Omid Mozaffari). The data underlying this figure can be found in https://zenodo.org/records/17080841.

The IUCN Red List and the PCI revealed similar results for several species (e.g., the Hula painted frog—*Latonia nigriventer* and Gyrfalcon—*Falco rusticolus*; Fig 5), whereas for others the results are vastly different (e.g., the gecko *Cyrtodactylus metropolis* and the yellow-crested cockatoo—*Cacatua sulphurea*; Fig 5). High variability in PCI scores within each IUCN Red List category (SD range: 0.06–0.14 standard deviation, on a scale from 0 to 1, Fig 2) reveals that species with very different future conservation priorities are potentially grouped in the same IUCN Red List category (Fig 2). Modifying the weights given to land use change, biological invasions, and human population density did not increase PCI scores’ correlation to IUCN Red List categories. This indicates that PCI may provide different information than the IUCN Red List regarding these projected threats (S1 Fig), which have been forecasted to be major drivers of biodiversity loss in the near future [22]. Unexpectedly, the PCI and the IUCN Red List had higher correlation when increasing the weight of climate change (S1 Fig), which indicates currently threatened species will be especially vulnerable to climate change. Our continuous index reveals new information about future conservation needs that can complement the IUCN Red List when prioritizing species and regions for conservation.

Our results show that species in the Near Threatened category (a “non-threatened” status) have PCI scores most similar to species in the “Vulnerable” category, considered threatened (Fig 2). Near Threatened Species may thus merit conservation attention similar to threatened species. In addition, Data Deficient and Non-Evaluated species, such as Val’s gundi (*Ctenodactylus vali*) and Papenfuss’s racerunner (*Eremias papenfussi*; Fig 5), have PCI scores that are, on average, similar to the scores of Endangered and Critically Endangered species (Fig 2). This result adds to accumulating evidence that Data Deficient and Non-Evaluated species are likely to be highly threatened [27–32]. The PCI can offer a way to prioritize the conservation of DD and NE species, which receive less conservation attention than species for which a threat category has been assigned [29], as it offers unique insights into threats these species are expected to face in coming decades. We suggest that conservation practitioners give due attention to conservation prioritization of Data Deficient, Non-Evaluated, and Near Threatened species, using complementary conservation prioritization tools such as the PCI.

A promising tool that might be useful in conjunction with the PCI is the IUCN Green Status of Species [33]. This future-focused score accounts for the future conservation dependence of a species and potential conservation gains both in the short and long term. Green Status assessments are currently available for only 115 species, including 78 terrestrial vertebrates, which makes it impractical to be incorporated in the PCI calculation now, but for the specific cases in which it is available, it could provide a more complete picture of the potential for conservation initiatives to address the threats highlighted by the PCI. For example, the Hainan black crested gibbon (*Nomascus hainanus*) is classified as Critically Endangered in the IUCN Red List and had the 41st highest PCI score among 5,658 mammal species for the year 2100, under scenario SSP 5.85. Its Green Status Assessment reveals the species is confined to a single protected area, due to land use change and overexploitation. It projects limited potential for increases in its population in the next 10 years, but a higher potential for population recovery in 100 years, based on a population viability analysis. The PCI, however, reveals that this species may also suffer from temperature extremes along all its range in the most extreme scenario for the year 2100. Thus, population recovery efforts could benefit from considering future climate projections, as climate change can affect this vulnerable species even in protected areas.

The PCI was most sensitive to the weighting of land use and human population density across future scenarios (S2 Fig). However, climate change became a dominant component in 2100 under the most extreme climate change scenario (SSP 5.85, S2 Fig). Under a milder emission scenario (SSP 2.45), average PCI scores in 2100 are much lower, especially in tropical regions (S8 Fig). This result highlights the importance of global efforts to mitigate carbon emissions, as climate change can become as great of a threat as land use change, if not addressed immediately. Our cluster analysis showed that species with small ranges will be more vulnerable to climate change, while species with less protected area coverage will be more vulnerable to land use change (S10 Fig). These results highlight that expanding the protected area network could be useful against land conversion, especially for species less tolerant to human presence, but this may be insufficient to protect small-range species, which may lose much of their ranges due to local climatic changes [34].

### Phylogenetic and geographic patterns

Reptiles are the vertebrate clade with the highest conservation priority under all future scenarios examined, adding to previous evidence that reptiles might be more threatened than currently depicted in the IUCN Red List [32]. Despite being the most species-rich vertebrate class, reptiles are relatively neglected in conservation research and actions [31,35,36]. Fossorial and cryptic reptiles such as amphisbaenians and blind snakes had high average PCI scores but a low proportion of threatened species in the IUCN Red List (S1 Data). These are poorly studied taxa, which usually lack sufficient information for formal threat assessments [37]. The PCI can offer a practical way to evaluate the conservation needs of hard to study groups, informing conservation actions that may prevent future threats to these neglected species. Reptiles had highest average PCI scores in western India (Western Ghats and Deccan Plateau) and in the Caribbean (Fig 3). Targeted conservation interventions in these areas have a high potential to prevent species loss for this vulnerable and neglected group.

The IUCN Red List shows amphibians as the most threatened class [3]. The PCI may underestimate amphibian threat status since it does not include the threat of chytridiomycosis, a fungal disease considered by many to be a major threatening process for amphibians [38]. However, amphibian mortality associated with fungal diseases is often exacerbated by other threats, such as climate change [39], which are accounted for in the PCI. Regardless, our results show convergent conservation needs between reptiles and amphibians. Our cluster analysis suggests that amphibians and reptiles will be more vulnerable to climate change than mammals and birds under the worst climate change scenario (SSP 5.85; S11 Fig). As in reptiles, families of cryptic and fossorial caecilians had the highest unrecognized conservation needs (S1 Data), and the Western Ghats also emerged as one of the highest priority regions for amphibians. However, unlike reptiles, amphibians showed high average PCI scores in some forests in China and Central Asia (S1 Data).

Islands located mainly in the Caribbean, Polynesia, and the Indian Ocean (including Socotra and Madagascar) had the highest average PCI scores for most vertebrate classes, except amphibians (Fig 4 and S2 Data). Islands have high degrees of endemism and phylogenetic uniqueness [40], and their vulnerable small-ranged [41] fauna is likely to suffer some of the worst consequences of climate change [42]. In fact, 75% of recorded tetrapod species extinctions have been on islands [43–45]. Specialized conservation interventions for the islands we identified as more likely to be threatened, could prevent the loss of ancient and unique evolutionary lineages [42]. Tropical forests in montane regions such as the Western Ghats also had high PCI scores. Montane regions may experience isolation patterns similar to islands: high altitudes offer strong geographical constraints, acting as “sky-islands” [46], which house unique evolutionary lineages [47,48] and are especially vulnerable to climate change [49,50]. Islands and montane regions will be particularly important for the conservation of bird biodiversity. The ecoregions with highest average PCI scores for birds were mostly in Polynesia and western India (Fig 3 and S3 Data). Additionally, bird families with high PCI scores but low proportion of threatened species were also endemic to islands and montane forests around the tropics, including Cuban warblers, rockjumpers, and modulatricids (S1 Data).

Although the ecoregions with the highest average PCI scores for mammals were also concentrated in islands, mainly in the Caribbean and Madagascar, the mammal families with high PCI scores and low proportion of threatened species were mostly from arid regions in Africa and Asia, including brush-tailed mice, gundis, hyenas, and hyraxes. This coincides with our geographical comparison between the PCI and the IUCN Red List (Fig 4), which suggests deserts, steppes, and savannas in Africa, Asia, South America, and North America will require a level of conservation priority not currently reflected in the IUCN Red List. Arid regions are traditionally neglected for conservation efforts and are likely to face severe threats from climate change and land use change in the near future [51].

Using the PCI to inform the prioritization of taxa and regions for conservation may increase efficiency in allocation of limited conservation resources. It can enable targeting species and regions that are predicted to be most affected by global changes in coming decades, potentially informing preventive, rather than palliative, investment in conservation. By design, the PCI is negatively correlated with protected area coverage, and thus highlights areas not currently receiving conservation attention. It also has the advantage of considering different future scenarios of climate change, land use change, and human population density, which may help differentiate types of intervention with higher impact. Our results suggest preventive conservation measures could have a high impact if targeted at arid regions and at insular and montane tropical forests, with particular focus on reptiles and cryptic species.

### Caveats and notes on implementation

In some cases, the IUCN Red List can include information on threats not captured by the PCI, as data on many important threat processes are not broadly available. For example, the Critically Endangered yellow-crested cockatoo (*C. sulphurea*; Fig 5) is heavily affected by pet trade, which was not accounted for in our study and thus has a low PCI score. Other species, such as the gecko *C. metropolis* (Fig 5), have a higher priority level in the PCI than in the IUCN Red List. Although this species has a very small range, and is located in the highly urbanized metropolitan area of Kuala Lumpur, it is reasonably well protected in the Batu caves, a cultural and religious site.

When calculating PCI scores, we consider only strict protected area categories (see Materials and methods), because the level of protection varies greatly between these categories. We provide data on protected area coverage, including less strict categories, in our R package, so users can incorporate it in the PCI.

The PCI reveals that the small range of *C. metropolis* will be highly susceptible to high levels of climate change: in the year 2100, under the most pessimistic scenario, *C. metropolis* is predicted to have all of its tiny range subject to extreme temperatures above historical records. In such scenarios, protected areas may be insufficient to protect this species. Our method is intended to complement the IUCN Red List, so we recommend that, in cases where the IUCN Red List and the PCI disagree substantially, conservation practitioners should examine threatening processes listed as most relevant under each method and use this complementary information to guide conservation actions. Comparisons made at the regional level, such as those displayed in Fig 4, highlight disagreements between the PCI and the IUCN Red List at larger scales, revealing broad patterns of information complementarity between the two methods, despite the limitations of either method at individual cases.

We recognize that the absence of global future projections for key threats, such as overexploitation or disease, can limit the application of the PCI. For species where these threats are critical, the PCI alone can be insufficient. In such cases, the PCI should be combined with other prioritization tools (e.g., the IUCN Red List) to ensure these threats are represented. Importantly, the PCI R package is designed so that additional information on additional threats can be incorporated as soon as global data becomes available. We try to approximate the threat of overexploitation using projections of human population density. Although human population density shows a strong correlation with overexploitation [52], it may underestimate the conservation needs of species affected mainly by this threat, but it may also reveal unrecognized threats which can interact with overexploitation, potentially compounding its threat [53]. Moreover, the variables chosen for our calculation do not consider local contexts or societal features, which may influence conservation priority levels. The PCI, as currently implemented, should be interpreted primarily as a measure of conservation prioritization in regard to the threats of climate change, land use change, biological invasions, and human population density, but users can incorporate any threat using our R package, if data are available.

A major challenge in designing and implementing unsupervised conservation prioritization schemes, such as the PCI, is determining the relative importance of threats and traits. We constructed the PCI and its associated online resources, to provide relative prioritization schemes customized to different sets of species, associated attributes, and weights, in different regions. We ensure users can implement any set of weights they deem appropriate for the context of their application. However, users should strive to determine a more appropriate set of weights *a priori*, based on empirical data, or on consensus in the scientific literature, instead of arbitrarily adjusting weights to achieve a desired result. Empirical data comparing the relative effect of threats could be used to set these weights in studies with more restricted taxonomic or geographic scope, for which this data could be available. We provide a weight optimization function with the PCI R package, which users can apply to optimize weights so PCI ranks match an external reference (e.g., the IUCN Red List). These optimized weights can then be applied to any set of species, including those not present in the reference prioritization rank or score. We do not apply this tool to our main results as the IUCN Red List is the only external reference available at global scale, and we make diverse comparisons between the PCI and the IUCN Red List.

We acknowledge that any prioritization framework, including the PCI and the IUCN Red List, involves arbitrary thresholds or weightings (e.g., range size thresholds for the IUCN criterion B, rates of decline for criteria A and C, and population sized for criteria C and D). To mitigate this, we make our weighting scheme explicit, provide sensitivity analyses, and offer users open tools to adapt weightings to the context of their use. This transparency allows users to understand where such arbitrariness may affect outcomes and adapt their decisions accordingly, rather than having them remain hidden. As long as the underlying data are available, PCI scores can easily be converted between different sets of weights, using our R package, making it simple to compare the results of different PCI implementations. We recommend that studies comparing different implementations of the PCI first convert values between different sets of weights used. We also recommend that users examine the sensitivity of results to different weight ranges, as we have done in our analyses (S1 and S2 Figs).

To maximize the longevity and accessibility of the PCI, we will maintain the tool through a publicly available GitHub repository (https://github.com/gabrielhoc/PCI), and archive it in the Comprehensive R Archive Network to ensure permanent access. As new global datasets, including other important threats such as overexploitation and diseases, become available, our R package and Shiny application will be updated to reflect the best information available. We will disseminate the PCI through collaborations with conservation organizations, workshops, and outreach efforts, which will be essential for ensuring that the PCI is adopted by a wider user community.

### Conclusions

The PCI offers a new tool to prioritize species and regions for conservation, by quantitatively estimating the expanding threat of human-induced environmental changes. The flexibility and ease of use of the PCI can provide a basis for planning the conservation of species groups currently lagging in the IUCN Red List assessments, such as plants and invertebrates, and generally complement the IUCN Red List across taxa. We show that future threats are likely to heavily affect most land vertebrates and ecoregions, but will be especially dire for reptiles, arid regions, insular, and montane tropical forests. These effects can be greatly diminished if greenhouse gas emissions are mitigated, and the protected area network is extended to currently neglected regions. We also show that species classified as Near Threatened and Data Deficient in the IUCN Red List, and those not yet evaluated, may need conservation prioritization similar to species classified under threatened categories. We hope our tool contributes to improve conservation planning in this crucial moment, when global changes are increasingly affecting our natural environments and daily lives.

## Materials and methods

### Index calculation

We included four main sources of threats in the calculation of the PCI: climate change, land use change, biological invasions, and human population density—across species ranges. Although not a direct threat, human population density is a driver of threats such as direct exploitation and pollution [7], and thus may serve as a proxy for these hard to measure threats. We also included five species-specific attributes that might modulate the effect of these threats: range size, body size, brood size, proportion of the range in protected areas, and tolerance to human-modified habitats. Although there are many other species attributes that may influence a species sensitivity to the threats, we limited our choice to variables that are widely available for most land vertebrate species. We focused on threats and traits more relevant for terrestrial organisms, as data on these are more readily available, but the index can be easily adapted to aquatic organisms by using our R package to input relevant threats and traits on the index’s calculation (see below). Taxonomic differences between datasets were standardized to match the species list in [5] using the R package “bdc” [54]. The PCI is a relative index, which varies from 0 (the score if a species is the least threatened with respect to every variable considered) to 1 (the score if the species is the most threatened with respect to every variable considered, compared to the other species included in the calculation).

To incorporate projected climate change effects on species, we used the proportion of each species’ range that will be subject to extreme temperature events [5]. These include higher intensity, frequency, or duration of extreme temperatures unprecedented in the species’ recent history, calculated for both 2050 and 2100 under either SSP 2.45 or 5.85 [5]. We chose to focus on extreme climatic events as they have more acute effects on biological systems than changes in mean climate [55,56]. To incorporate projected land use change effects on species, we used the proportion of a species range which will be under anthropogenic land uses (cropland, pasture, and urban areas). We used land use classes projections from the Land Use Harmonization 2 (0.25 degree resolution, [57] for 2050, and 2100 under SSP 2.45 and 5.85. To incorporate projected trends in human population density, we calculated population density (persons/km^2^) across a species range for the same future years and scenarios [58]. We calculated the percentage of each species range under high or very high future biological invasion threat, using the classification by Early and colleagues 2016 [59] for 2100 under scenario A2. We use the same value of biological invasion threat for every future scenario, as it was the only scenario available. Range sizes (km^2^) were obtained from the Global Assessment of Reptile Distributions (GARD) for reptiles [36], BirdLife International for birds (www.birdlife.org), and the IUCN for amphibians and mammals (www.iucnredlist.org). We calculated the exposure of species to future threats within their current ranges, as projecting future ranges is beyond the scope of this paper and could limit the number of species to which the method could be applied. However, range shifts may substantially change threat levels [20,60], and users interested in using threat exposure metrics based on future projected ranges can easily do so using our R package. We use future states of land use and human population density in our calculations, as we think it better reflects the exposure of species at that point in time. However, we also include in our R package metrics of current land use and human population density, so users may calculate rates of change for these variables, if they wish to.

For the body size metric, we used maximum adult body mass (grams) obtained from the GARD database for reptiles (https://gardinitiative.org, [61]) and the Global Amphibian Biodiversity Project for amphibians (https://amphibianbiodiversity.org), and mean adult body mass (grams) from AVONET for birds [62] and COMBINE for mammals [63]. Body size data were supplemented by imputed values for missing data (13% for amphibians, 20% for birds, 2% for reptiles, 13% for mammals) using phylogenetic imputation in the R package Rphylopars [64] with previously published phylogenies [65–69]. Likewise, brood size data [61,70] were supplemented by imputed values for missing data (70% for amphibians, 53% for birds, 46% for reptiles, 39% for mammals). For species with missing body size data (1,845) and missing brood size data (3,891) not represented in the phylogenies, we input them with the median values for these variables in their respective families. We further calculated the proportion of a species range which is covered by an IUCN protected area in categories I to IV (which are set aside strictly for biodiversity conservation [71]), obtained from the September 2022 version of the World Database on Protected Areas [72]. To incorporate intolerance of species to human-modified habitats, we used habitat preference information in the IUCN Red List [3]. Species for which artificial environments (those preceded by the tag “Artificial” in the IUCN habitat classification) were classified as “suitable” received a score of 0, species for which artificial environments were classified as “marginal” received a score of 0.5, and the remaining species (those with no “Artificial” tag in their IUCN habitat classification and those not in the IUCN Red List) received a score of 1. Although population size is an important metric to assess vulnerability, it is not available for most species. Some of the traits included in our calculations are correlated to population size and may approximate its effects, such as range size and body size [73]. The PCI is not designed to assess species already severely depleted in the past. We stress that, in such cases, the PCI should be used as complementary to frameworks such as the IUCN Red List, which focus on past and present threats. Used together, these approaches can provide a more complete picture of conservation priorities.

We calculated the PCI for 10,892 reptile, 5,658 mammal, 10,078 bird, and 6,932 amphibian species, for two future years (2050 and 2100), under two SSP (i.e., land use and climatic scenarios: SSP 2.45 predicting relatively mild climate changes and SSP 5.85 predicting more severe/drastic warming). Since the distributions of most variables in the PCI are right-skewed and vary by orders of magnitude (e.g., range size, body size), we added one to the values of each datum and log-transformed it. We then scaled each variable between 0 and 1, using a *min-max normalization* function ($f_{s}$, Equation 1).


$$f_{s}(x)=\frac{x–min(x)}{max(x)–min(x)}$$ (1)

In which *x* is a vector being scaled. We then calculated the weighted interaction products (*r*_*i*_) for each threat variable *i* (Equation 2):


$$\begin{array}{c} r_{i}=\sum {\mathrm{\backslash nolimits}}_{j=\text{1}}^{m}\left(t_{i}⊙\left(v_{j}z_{i,j}\right)\right) \end{array}$$ (2)

where *t*_*i*_ is a vector containing the scaled values of threat variable *i* for all species, *v*_*j*_ is a vector containing the values of interacting variable *j* for all species, *m* is the number of interacting variables, and *z*_*i,j*_ is the weight for the interaction between threat variable *i* and interacting variable *j.* The symbol ⊙ denotes element-wise multiplication. We then calculated the PCI as the weighted arithmetic mean of the scaled interaction products for each combination of threat variables (Equation 3):


$$p=f_{s}\left(\frac{\sum_{i=\text{1}}^{n}\left(f_{s}\left(r_{i}\right)w_{i}\right)}{\sum_{i=\text{1}}^{n}w_{i}}\right)$$ (3)

In which *p* is the vector of PCI scores for all species, *n* is the total number of threat variables (we used *n* = 7, but users can input other numbers of variables using our dedicated R package—see below), and *w*_*i*_ is the weight of threat variable *i*. The index’s calculation is illustrated in Fig 1.

The weighting scheme described above allows users to adjust the index according to which threatening process they consider more important for their specific taxa or location. This approach also enables users of the PCI to further adjust the interacting variables that might have a higher effect on each threatening process. Weights are scaled during the calculation, so weights with different absolute values may produce the same results, if they have the same proportion between each other. We have included a weight optimization feature in our R package, which allows the estimation of weights so PCI scores match an external reference (such as IUCN Red List categories) as closely as possible. This can be used, for example, to calibrate weights using a subset of species for which another ranking or conservation priority index is available, and then use these weights to calculate the PCI for the remainder of the set of species of interest. We did not use this feature in our geographical and taxonomic representations, instead assigning the same weight to each variable, as we aimed to produce scores that are independent of the IUCN Red List, so we can compare prioritization schemes based on the PCI and on the IUCN Red List.

### Complementarity of the PCI and the IUCN Red List

We conducted a series of analyses to compare threat rankings of the PCI and the IUCN Red List. We calculated Spearman’s correlation coefficient between PCI scores and IUCN Red List categories, ranked from more threatened to less threatened, for each future scenario, and examined the distribution of PCI scores for each IUCN Red List category to evaluate the congruencies and divergences between these two indexes’ scores. We repeated this analysis using only species categorized as threatened by the IUCN Red List under criteria A3 and E criteria, which are solely based on future threats (310 species), together with all non-threatened species (under the Near Threatened and Least Concern categories). We further fitted a Generalized Linear Model [74], using a binomial distribution and logit link function, between the proportion of threatened species and the average PCI scores for the species in each ecoregion of the world [75]. We examined the geographical distribution of the models’ residuals, to identify areas designated as containing proportionally many threatened species based on either PCI scores or the IUCN Red List. We investigated the influence of weighting of factors in the PCI on the correlation between PCI scores and IUCN Red List categories, by recalculating the PCI after assigning weights ranging between 0 and 10 to each variable consecutively, while holding the other variables at weight = 1, and recalculating the correlation coefficient between PCI scores and IUCN Red List categories.

### Phylogenetic and spatial patterns

We compared the distribution of PCI scores between land vertebrate classes and for each ecoregion of the world [75], in each of the future scenarios. For the ecoregion analysis, we calculated the average and total (sum) of PCI scores of all species whose range overlaps each ecoregion. We also calculated the average PCI score for each land vertebrate family and mapped these values across a phylogenetic tree for each class [65–69], assigning to each node the average PCI score of its descendants.

### Patterns in the PCI components

Additionally, we explored the distribution of our threat and interacting variables, using hierarchical clustering (with function “hclust,” from package “stats” [74], and a sensitivity analysis of the weights for each variable in the PCI. For the sensitivity analysis, we recalculated the PCI after assigning a weight of 0.1, 1, or 10 to each variable consecutively, while holding the other variables at weight of 1, and then recalculating the PCI so we could assess the effect of each variable on the distribution of the PCI. All analyses were performed in R 4.2.1 [25].

## Supporting information

S1 Table Spearman’s correlation of PCI scores and IUCN Red List categories of land vertebrates in four future scenarios. We considered only species in the Least Concern, Near Threatened, Vulnerable, Endangered, and Critically Endangered categories, which were respectively converted in a numeric scale, from 1 to 5, from less threatened to more threatened. We repeated the analysis, including only species designated as threatened under criteria A3 and E and species under non-threatened categories (NT and LC).
(DOCX)

S2 Table Mean (± standard deviation) of Proactive Conservation Index (PCI) for four land vertebrate classes, in 2050 and 2100, under two future scenarios. Analyses of variance for each year and scenario combination had 3 degrees of freedom, *F* > 900, and *p* < 0.0001.
(DOCX)

S1 Fig Sensitivity of Spearman’s correlation between Proactive Conservation Index (PCI) and IUCN Red List categories to differential weighting of the variables used in the calculation of PCI. The data underlying this figure can be found in https://zenodo.org/records/17080841.
(DOCX)

S2 Fig Distribution of log-transformed Proactive Conservation Index (PCI) scores for land vertebrates in four future scenarios, under three weighting schemes for the variables used in the index’s calculation. We changed the weights of each variable consecutively to 0.1, 1, and 10, while holding the weights for other variables at 1, and recalculated the index for each weight combination. The data underlying this figure can be found in https://zenodo.org/records/17080841.
(DOCX)

S3 Fig Distribution of Proactive Conservation Index (PCI) across the four land vertebrate classes, in two future Shared Socioeconomic Pathway (SSP) scenarios during 2050 and 2100. The data underlying this figure can be found in https://zenodo.org/records/17080841.
(DOCX)

S4 Fig Average Proactive Conservation Index for amphibian families in the year 2100 under SSP 5.85. The data underlying this figure can be found in https://zenodo.org/records/17080841.
(DOCX)

S5 Fig Average Proactive Conservation Index for bird families in the year 2100 under SSP 5.85. The data underlying this figure can be found in https://zenodo.org/records/17080841.
(DOCX)

S6 Fig Average Proactive Conservation Index for mammal families in the year 2100 under SSP 5.85. The data underlying this figure can be found in https://zenodo.org/records/17080841.
(DOCX)

S7 Fig Average Proactive Conservation Index for reptile families in the year 2100 under SSP 5.85. The data underlying this figure can be found in https://zenodo.org/records/17080841.
(DOCX)

S8 Fig Average Proactive Conservation Index for land vertebrates, across the ecoregions of the world, in four future scenarios. Shapefile for ecorregions was obtained from Olson and colleagues 2001 [75]. The data underlying this figure can be found in https://zenodo.org/records/17080841.
(DOCX)

S9 Fig Within-Cluster Sum of Squares for different numbers of clusters grouping land vertebrate species in respect to variables used in the calculation of the Proactive Conservation Index. The data underlying this figure can be found in https://zenodo.org/records/17080841.
(DOCX)

S10 Fig Distribution of variables used to calculate Proactive Conservation Index in four clusters of land vertebrate species grouped by hierarchical clustering. The data underlying this figure can be found in https://zenodo.org/records/17080841.
(DOCX)

S11 Fig Distribution of land vertebrate species across four clusters of land vertebrate species grouped by hierarchical clustering in respect to variables used in the calculation of Proactive Conservation Index. The data underlying this figure can be found in https://zenodo.org/records/17080841.
(DOCX)

S1 Data Average Proactive Conservation Index (PCI) scores and proportion of threatened species according to the IUCN Red List for each family of terrestrial vertebrates. Also included are the number of species in each family and in which quartile the average PCI scores and proportion of threatened species are in relation to all families in the same class.
(XLSX)

S2 Data Average Proactive Conservation Index (PCI) scores for terrestrial vertebrates in each ecoregion of the world. Average scores are displayed for each terrestrial vertebrate class (mammals, birds, reptiles, and amphibians) and for all classes combined, for future projections in the years 2050 and 2100, under scenarios SSP 2.45 and SSP 5.85.
(XLSX)

S3 Data Average Proactive Conservation Index (PCI) scores and proportion of threatened species according to the IUCN Red List for terrestrial vertebrates in each ecoregion of the world. Average scores are displayed for all terrestrial vertebrate classes (mammals, birds, reptiles, and amphibians) combined, for the year 2100 under scenario SSP 5.85. Also displayed are the residuals and log-residuals of a regression between average PCI scores and proportion of threatened species.
(XLSX)

## Abbreviations:

GARD: Global Assessment of Reptile Distributions
IUCN: International Union for Conservation of Nature
PCI: Proactive Conservation Index
SSP: shared socioeconomic pathways
