# Supplementary material for: The future-focused Proactive Conservation Index highlights unrecognized global priorities for vertebrate conservation
Source: PLoS Biol. 2025 Oct 21;23(10):e3003422. doi: 10.1371/journal.pbio.3003422 (PMC12539808; doi:10.1371/journal.pbio.3003422)
Supplement: S1 Table — We considered only species in the Least Concern, Near Threatened, Vulnerable, Endangered, and Critically Endangered categories, which were respectively converted in a numeric scale, from 1 to 5, from less threatened to more threatened. We repeated the analysis, including only species designated as threatened under criteria A3 and E and species under non-threatened categories (NT and LC). (DOCX) [file pbio.3003422.s001.docx]

**S1 Table. Spearman’s correlation of PCI scores and IUCN Red List categories of land vertebrates in four future scenarios.** We considered only species in the Least Concern, Near Threatened, Vulnerable, Endangered and Critically Endangered categories, which were respectively converted in a numeric scale, from 1 to 5 from less threatened to more threatened. We repeated the analysis including only species designated as threatened under criteria A3 and E and species under non-threatened categories (NT and LC).

| **Year** | **Scenario** | **Spearman’s rho** | **S-statistic** | **p-value** |
| --- | --- | --- | --- | --- |
| All species | | | | |
| 2050 | SSP 2.45 | 0.318 | 2.770*10^12^ | <0.001 |
| 2050 | SSP 5.85 | 0.367 | 2.573*10^12^ | <0.001 |
| 2100 | SSP 2.45 | 0.338 | 2.691*10^12^ | <0.001 |
| 2100 | SSP 5.85 | 0.370 | 2.563*10^12^ | <0.001 |
| Species under A3 and E criteria only + NT and LC | | | | |
| 2050 | SSP 2.45 | 0.127 | 1.646*10^12^ | <0.001 |
| 2050 | SSP 5.85 | 0.156 | 1.593*10^12^ | <0.001 |
| 2100 | SSP 2.45 | 0.136 | 1.630*10^12^ | <0.001 |
| 2100 | SSP 5.85 | 0.157 | 1.591*10^12^ | <0.001 |
