# Supplementary material for: The future-focused Proactive Conservation Index highlights unrecognized global priorities for vertebrate conservation
Source: PLoS Biol. 2025 Oct 21;23(10):e3003422. doi: 10.1371/journal.pbio.3003422 (PMC12539808; doi:10.1371/journal.pbio.3003422)

**S2 Table. Mean (± standard deviation) of Proactive Conservation Index (PCI) for four land vertebrate classes, in 2050 and 2100, under two future scenarios.** Analyses of variance for each year and scenario combination had 3 degrees of freedom, F > 900, and p < 0.0001.

| **Year** | **Scenario** | **Birds** | **Amphibians** | **Mammals** | **Reptiles** |
| --- | --- | --- | --- | --- | --- |
| 2050 | SSP 2.45 | 0.095 (0.064) | 0.107 (0.093) | 0.138 (0.087) | 0.148 (0.104) |
| 2050 | SSP 5.85 | 0.102 (0.068) | 0.123 (0.101) | 0.153 (0.094) | 0.167 (0.114) |
| 2100 | SSP 2.45 | 0.099 (0.066) | 0.117 (0.092) | 0.147 (0.090) | 0.157 (0.109) |
| 2100 | SSP 5.85 | 0.123 (0.076) | 0.151 (0.113) | 0.186 (0.105) | 0.197 (0.123) |

**Figure S1. Sensitivity of Spearman’s correlation between Proactive Conservation Index (PCI) and IUCN Red List categories to differential weighting of the variables used in the calculation of PCI.**  The data underlying this Figure can be found in https://zenodo.org/records/17080841


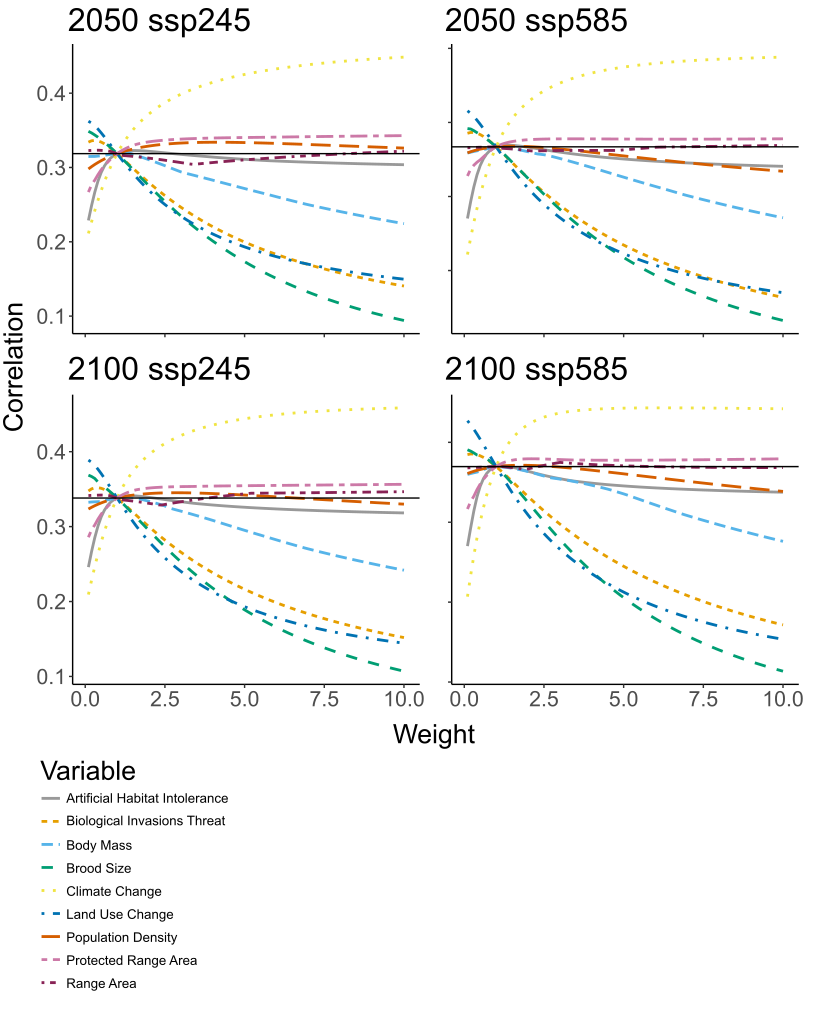


**Figure S2. Distribution of log-transformed Proactive Conservation Index (PCI) scores for land vertebrates in four future scenarios, under three weighting schemes for the variables used in the index’s calculation.** We changed the weights of each variable consecutively to 0.1, 1 and 10, while holding the weights for other variables at 1, and recalculated the index for each weight combination. The data underlying this Figure can be found in https://zenodo.org/records/17080841


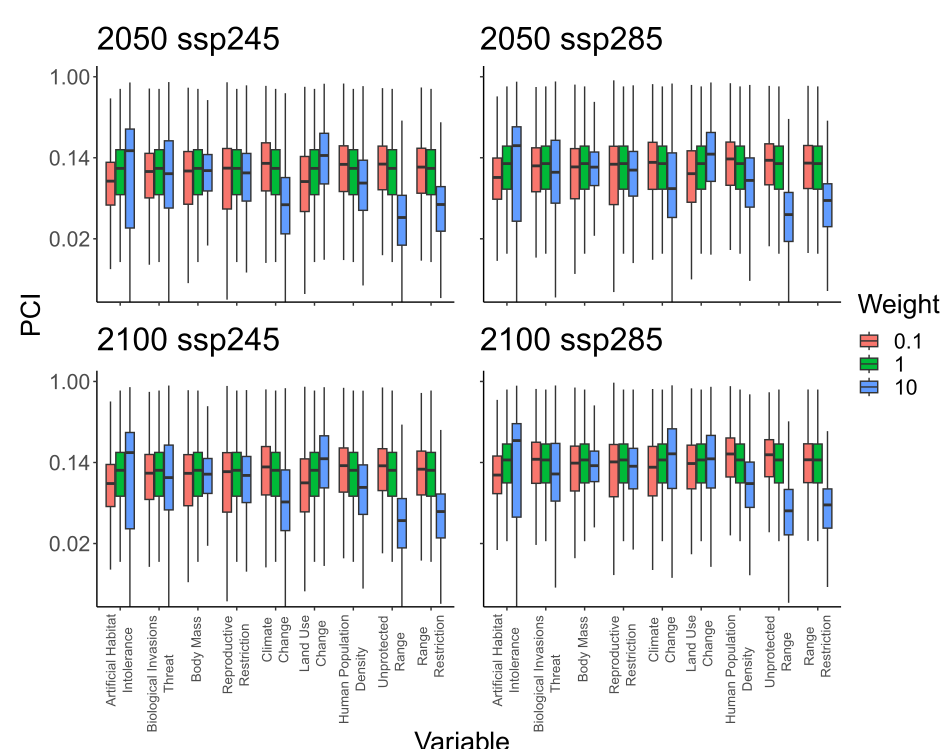


**Figure S3. Distribution of Proactive Conservation Index (PCI) across the four land vertebrate classes, in two future Shared Socioeconomic Pathway (SSP) scenarios during 2050 and 2100.** The data underlying this Figure can be found in https://zenodo.org/records/17080841


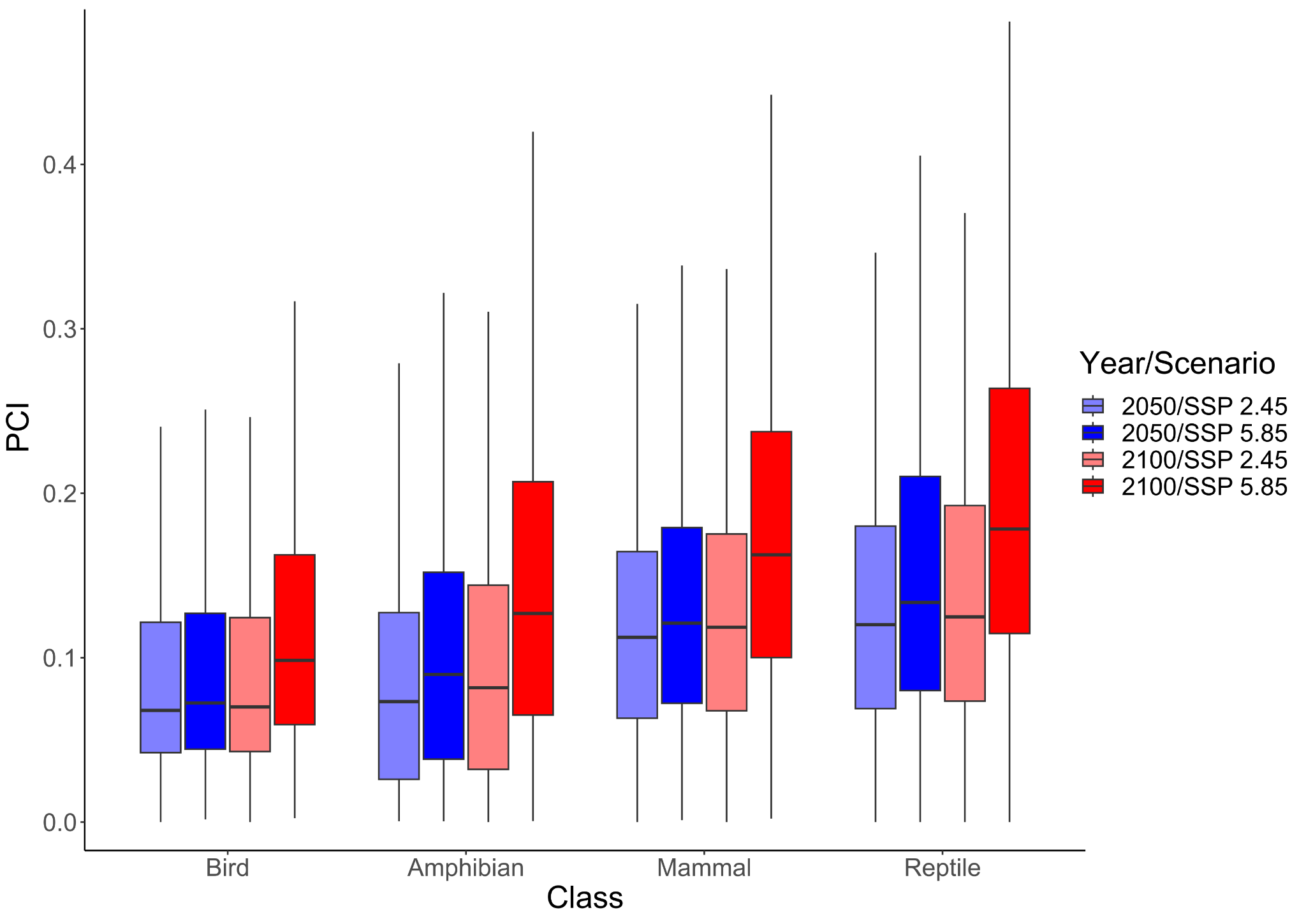


**Figure S4. Average Proactive Conservation Index for amphibian families in the year 2100 under SSP5.85.** The data underlying this Figure can be found in https://zenodo.org/records/17080841


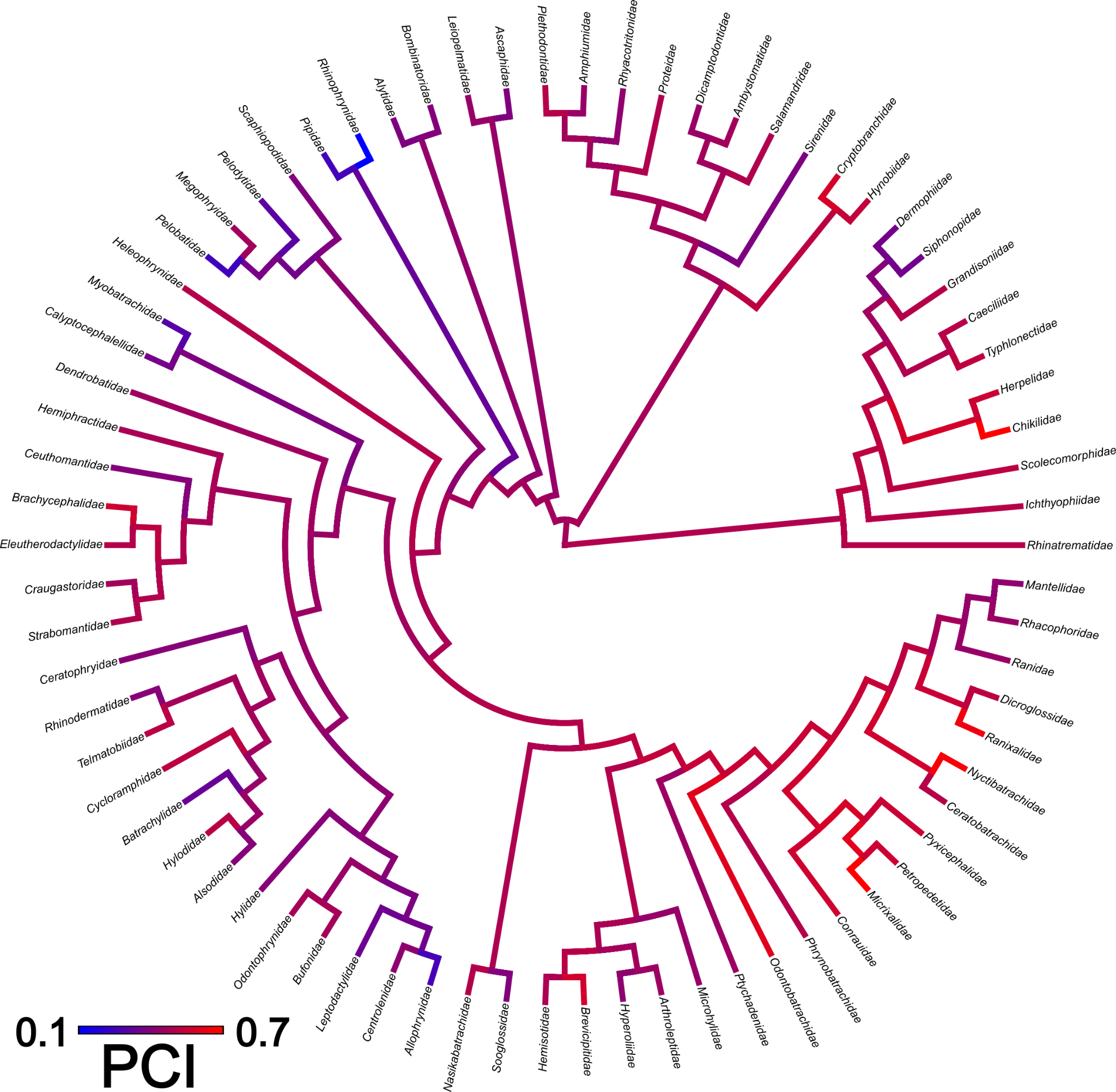


**Figure S5. Average Proactive Conservation Index for bird families in the year 2100 under SSP5.85.** The data underlying this Figure can be found in https://zenodo.org/records/17080841


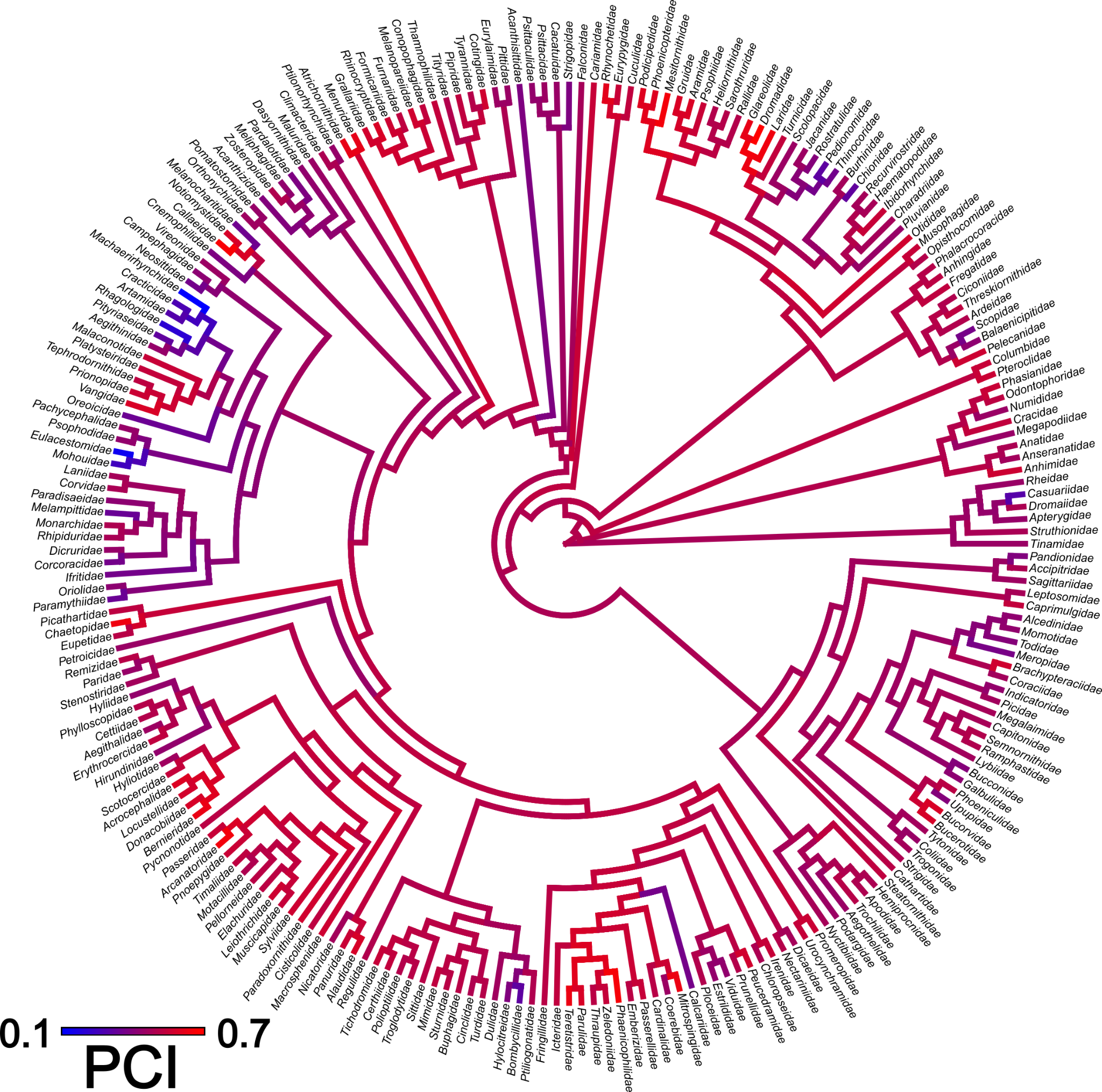


**Figure S6. Average Proactive Conservation Index for mammal families in the year 2100 under SSP5.85.** The data underlying this Figure can be found in https://zenodo.org/records/17080841


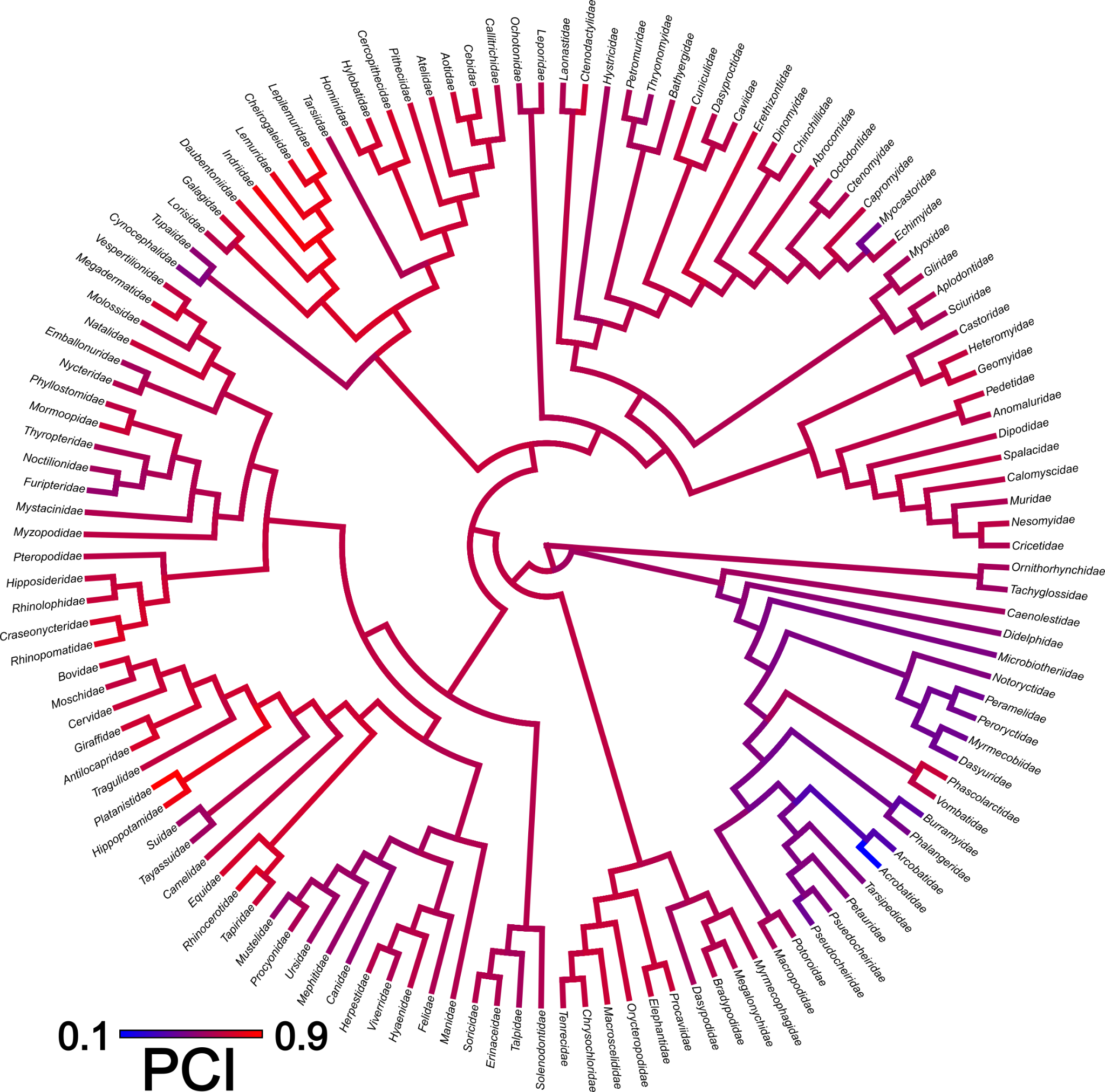


**Figure S7. Average Proactive Conservation Index for reptile families in the year 2100 under SSP5.85.** The data underlying this Figure can be found in https://zenodo.org/records/17080841


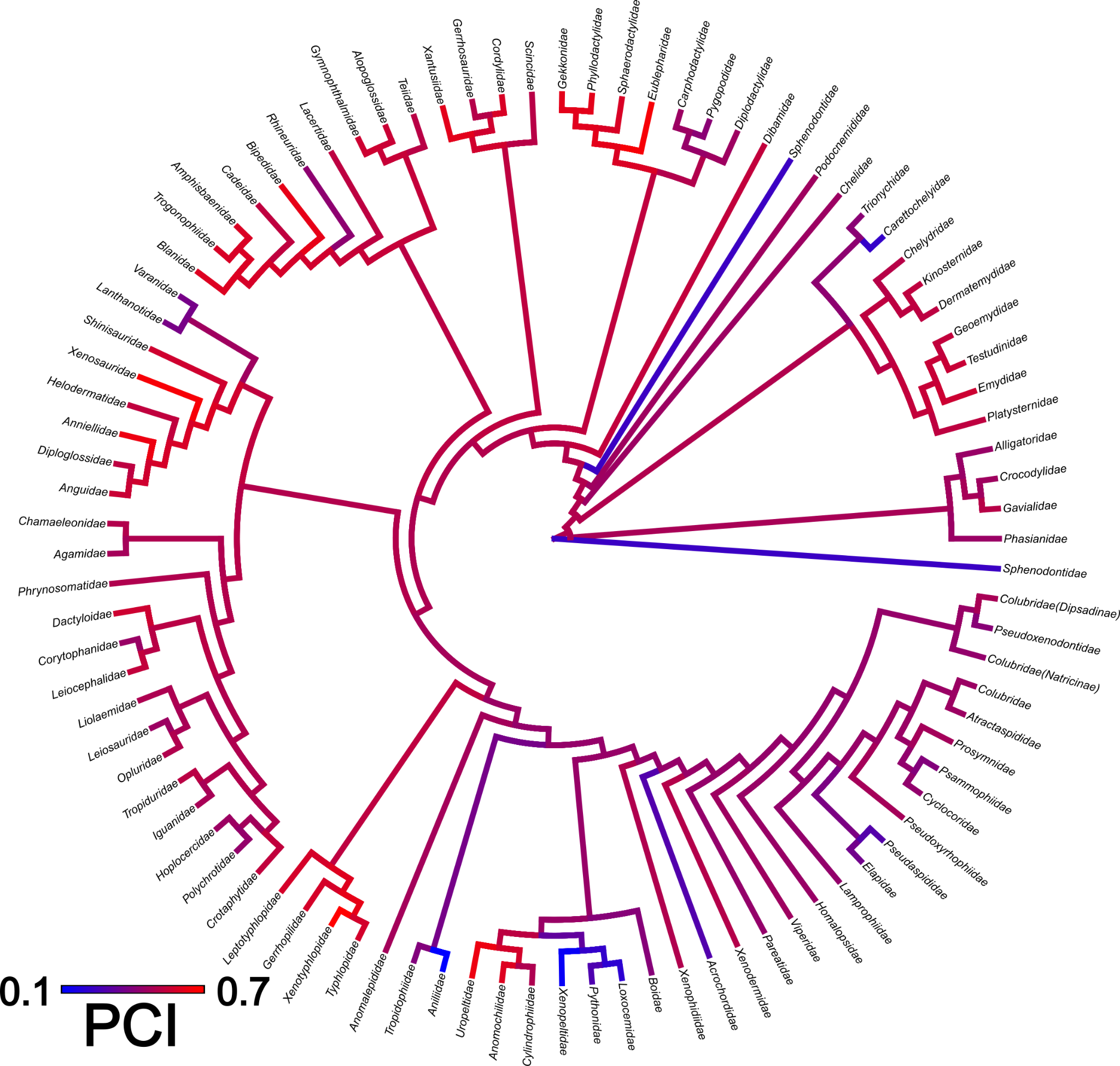


**Figure S8. Average Proactive Conservation Index for land vertebrates, across the ecoregions of the world, in four future scenarios.** Shapefile for ecorregions was obtained from Olson et al 2001 [75]. The data underlying this Figure can be found in https://zenodo.org/records/17080841


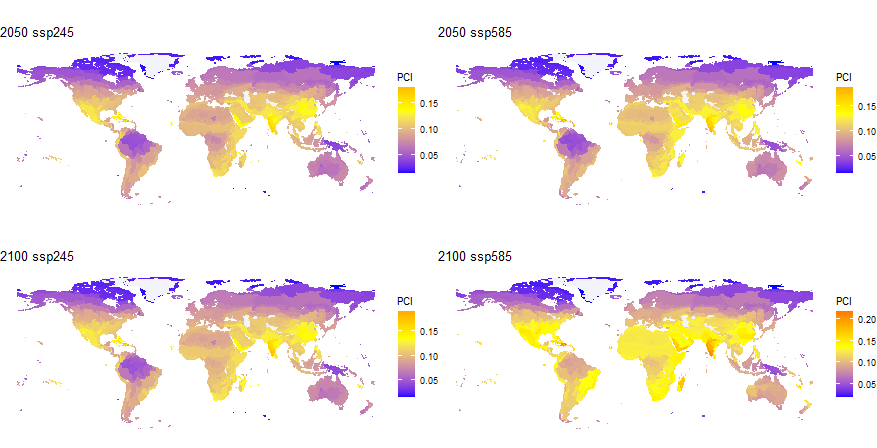


**Figure S9. Within-Cluster Sum of Squares for different numbers of clusters grouping land vertebrate species in respect to variables used in the calculation of the Proactive Conservation Index.** The data underlying this Figure can be found in https://zenodo.org/records/17080841


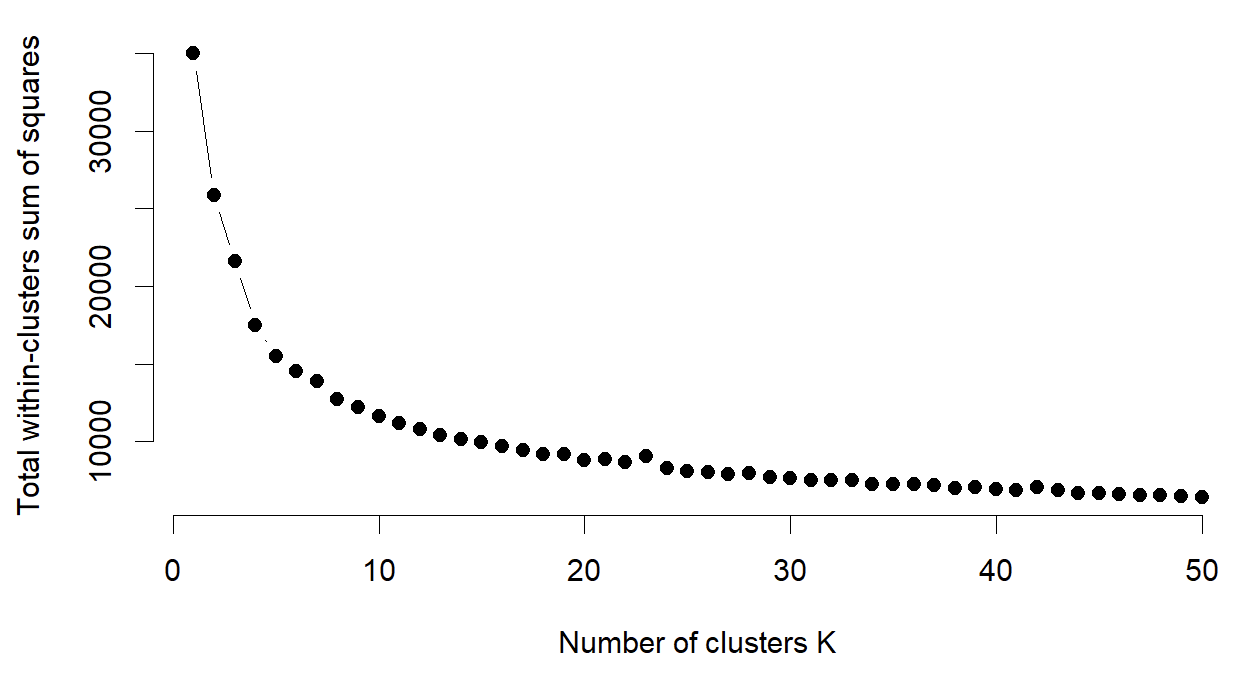


**Figure S10. Distribution of variables used to calculate Proactive Conservation Index in four clusters of land vertebrate species grouped by hierarchical clustering.** The data underlying this Figure can be found in https://zenodo.org/records/17080841


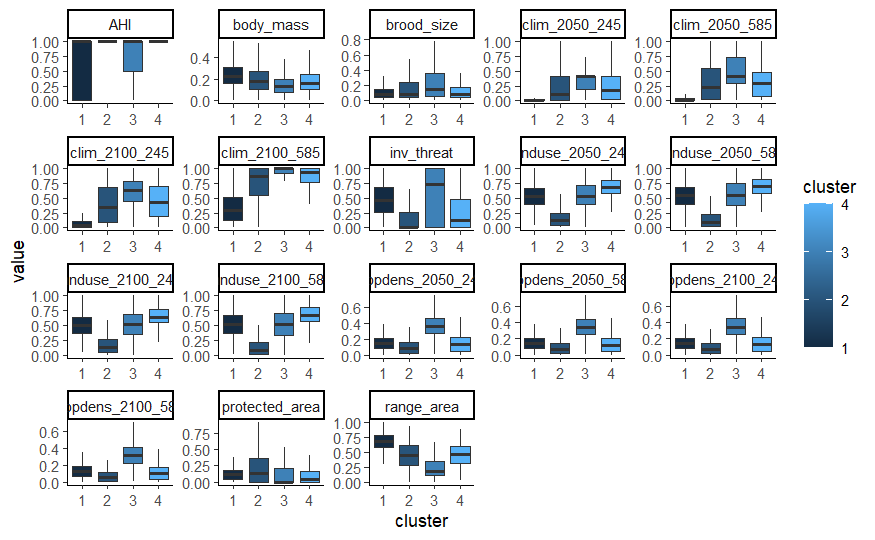


**Figure S11. Distribution of land vertebrate species across four clusters of land vertebrate species grouped by hierarchical clustering in respect to variables used in the calculation of Proactive Conservation Index.** The data underlying this Figure can be found in https://zenodo.org/records/17080841


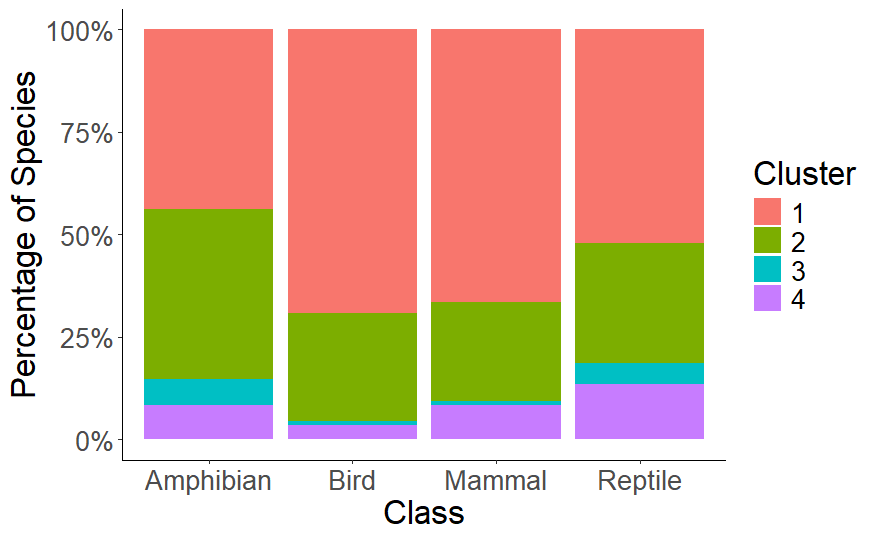

Supplement: S2 Table — Analyses of variance for each year and scenario combination had 3 degrees of freedom, F > 900, and p < 0.0001. (DOCX) [file pbio.3003422.s002.docx]
