# Supplementary material for: The future-focused Proactive Conservation Index highlights unrecognized global priorities for vertebrate conservation
Source: PLoS Biol. 2025 Oct 21;23(10):e3003422. doi: 10.1371/journal.pbio.3003422 (PMC12539808; doi:10.1371/journal.pbio.3003422)

**S1 Fig. Sensitivity of Spearman’s correlation between Proactive Conservation Index (PCI) and IUCN Red List categories to differential weighting of the variables used in the calculation of PCI.**  The data underlying this Figure can be found in https://zenodo.org/records/17080841


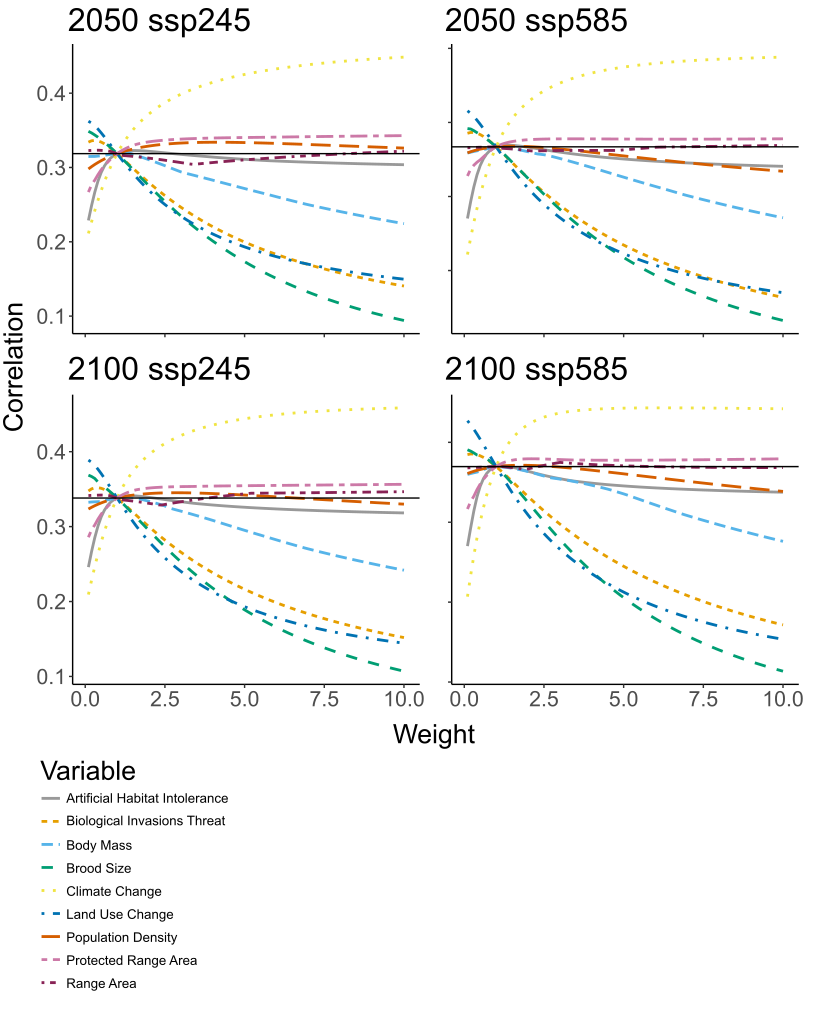

Supplement: S1 Fig — The data underlying this figure can be found in https://zenodo.org/records/17080841. (DOCX) [file pbio.3003422.s003.docx]
