# Supplementary material for: The future-focused Proactive Conservation Index highlights unrecognized global priorities for vertebrate conservation
Source: PLoS Biol. 2025 Oct 21;23(10):e3003422. doi: 10.1371/journal.pbio.3003422 (PMC12539808; doi:10.1371/journal.pbio.3003422)

**S2 Fig. Distribution of log-transformed Proactive Conservation Index (PCI) scores for land vertebrates in four future scenarios, under three weighting schemes for the variables used in the index’s calculation.** We changed the weights of each variable consecutively to 0.1, 1 and 10, while holding the weights for other variables at 1, and recalculated the index for each weight combination. The data underlying this Figure can be found in https://zenodo.org/records/17080841


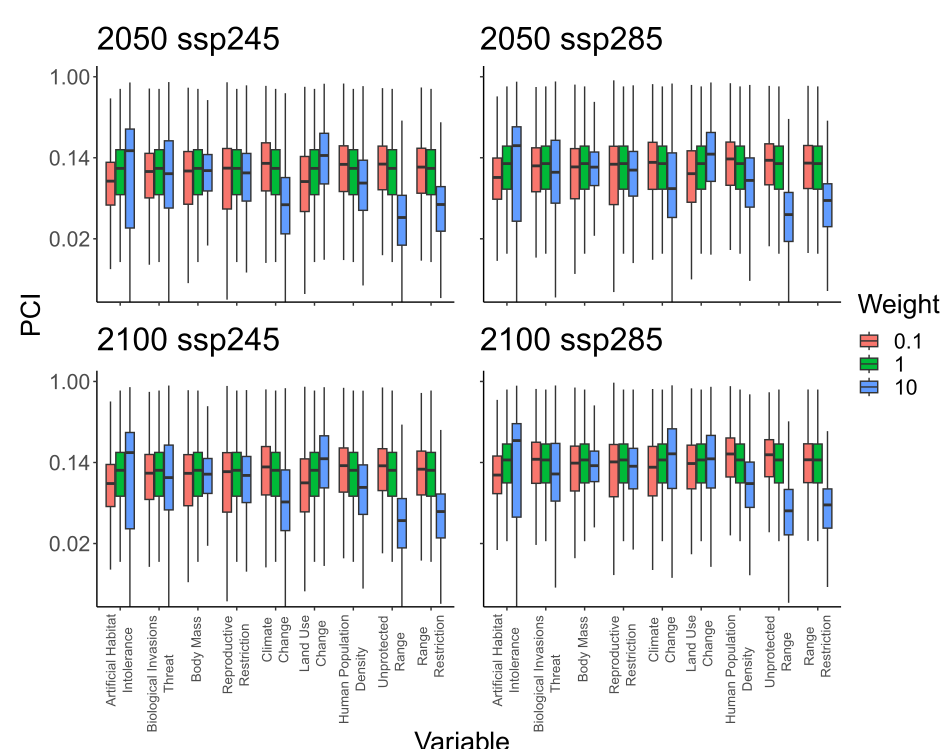

Supplement: S2 Fig — We changed the weights of each variable consecutively to 0.1, 1, and 10, while holding the weights for other variables at 1, and recalculated the index for each weight combination. The data underlying this figure can be found in https://zenodo.org/records/17080841. (DOCX) [file pbio.3003422.s004.docx]
