# Supplementary material for: The future-focused Proactive Conservation Index highlights unrecognized global priorities for vertebrate conservation
Source: PLoS Biol. 2025 Oct 21;23(10):e3003422. doi: 10.1371/journal.pbio.3003422 (PMC12539808; doi:10.1371/journal.pbio.3003422)

**S5 Fig. Average Proactive Conservation Index for bird families in the year 2100 under SSP5.85.** The data underlying this Figure can be found in https://zenodo.org/records/17080841


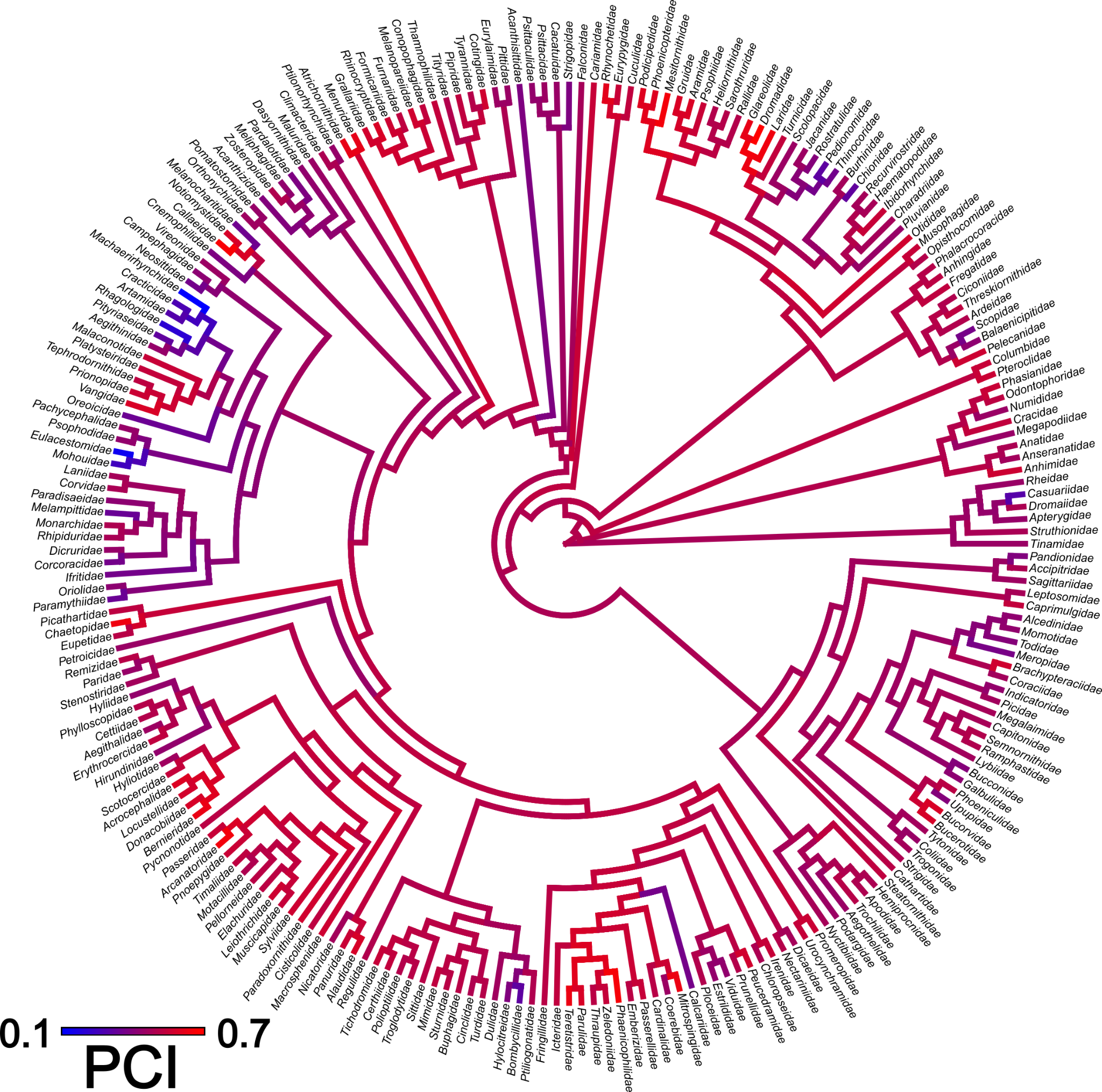


**Figure S11. Distribution of land vertebrate species across four clusters of land vertebrate species grouped by hierarchical clustering in respect to variables used in the calculation of Proactive Conservation Index.** The data underlying this Figure can be found in https://zenodo.org/records/17080841


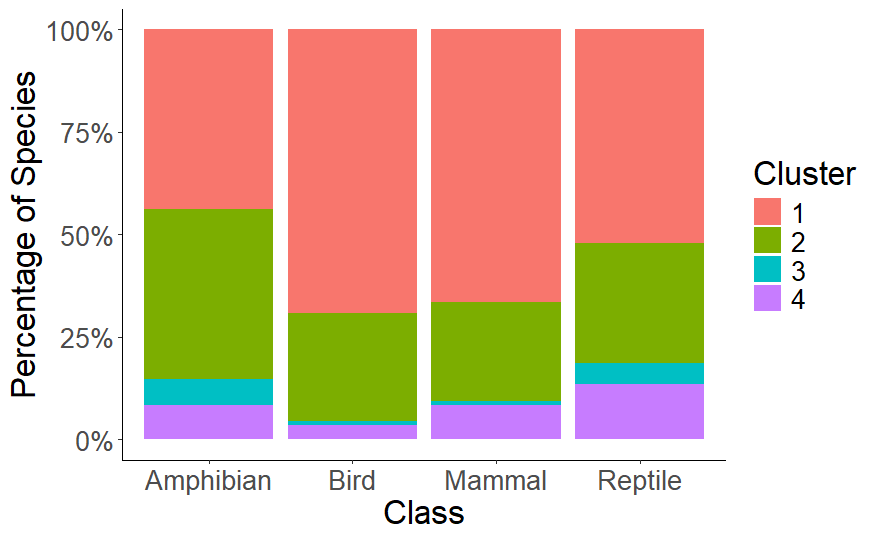

Supplement: S5 Fig — The data underlying this figure can be found in https://zenodo.org/records/17080841. (DOCX) [file pbio.3003422.s007.docx]
